# Tumor Protective Activity of CD4+ but Not of CD8+ T Cells in DNA-Vaccinated Mice Challenged with bcr-abl-Transformed Cells

**DOI:** 10.1155/2013/923107

**Published:** 2013-11-21

**Authors:** Martina Petráčková, Vincent Lučanský, Vladimír Vonka

**Affiliations:** Institute of Hematology and Blood Transfusion, Department of Experimental Virology, U Nemocnice 1, 12820 Prague 2, Czech Republic

## Abstract

In the recent past, it has repeatedly been reported that CD4 cells play an important role in the immunology of chronic myeloid leukaemia. It was therefore of interest to test their activity in an animal model using bcr-abl-transformed cells. BALB/c mice were four times immunized with a DNA vaccine carrying the bcr-abl fusion gene. Two weeks after the last vaccine dose, the animals were challenged with syngeneic bcr-abl-transformed 12B1 cells which form solid tumors after subcutaneous administration. At the time of challenge, animals were treated with antibodies against the CD8+ T cells or CD4+ T cells. The efficacy of the depletion was monitored and found highly effective. All nonimmunized animals developed tumors. All animals untreated with the antibodies as well as those in which CD8+ T cells had been depleted, were fully protected against the challenge. On the other hand, almost all mice treated with anti-CD4+ antibody developed tumors. These results strongly suggested that the CD4+ T cells acted as effectors in the present system.

## 1. Introduction

It is generally accepted that specifically activated CD8+ T cells play the most important role in immunological tumor rejection and a great majority of immunotherapeutic studies have been focusing on them. In the recent past, it has repeatedly been demonstrated that the CD4+ T cell response is polyfunctional and there has been growing evidence that a subpopulation of CD4+ T cells can mediate an efficient antitumor activity in some systems (for review see [1, 2]). Activated CD4+ T cells may partner with many different types of host cells to clear the tumor indirectly by secretion of vast array of cytokines. These cytokines react with and activate distinct classes of cells including macrophages, eosinophils, NK cells, and B cells and can induce antitumor effect independent of CD8+ T cells [3]. 

Evidence of direct cytotoxic role for CD4+ T cells has also been presented [4, 5]. Cytotoxic CD4+ T cells have been detected in peripheral blood of subjects suffering from various diseases such as those caused by viruses (HIV, CMV, and EBV) (for review see [6]) and chronic inflammatory diseases as rheumatoid arthritis [7] and B-cell chronic lymphocytic leukaemia [8]. It has been shown that their cytotoxic effect has been based on perforin-dependent pathway [9] and/or on the Fas-FasL-mediated apoptosis [10], and it has been suggested that the lytic activity of CD4+ T cells is likely HLA class II restricted, at least in some instances. 

In our previous study, we have shown that it is possible to induce solid immunity against the bcr-abl-transformed mouse cells by immunization with a DNA vaccine carrying the bcr-abl fusion gene [11]. It was the purpose of the present study to identify effector cells responsible for the protection.

## 2. Methods

### 2.1. Cells

The mouse (BALB/c) 12B1 cells transformed by human bcr-abl fusion gene [12] were used in the present experiments. They were obtained through the courtesy of E. Katsanis (University of Arizona, Tucson, AZ, USA) and cultivated as described previously [13]. In brief, they induce leukaemia-like disease after intravenous administration and solid tumors after subcutaneous administration. They express MHC class I molecules and are of pre-B origin. 

### 2.2. Animal Experiments

Seven-week old female BALB/c mice were obtained from Charles Rivers, Germany. All animal experiments were carried out in accordance with the Guidelines for Animal Experimentation valid in the Czech Republic. The human pBSC/bcr-abl [11] or the empty pBSC [14] expression plasmids were used as DNA vaccines. DNA cartridges were prepared according to the manufacturer's instructions (Helios Gene Gun System, Bio-Rad, Hercules, USA). Using the gene gun, we applied 1 *μ*g of DNA on gold particles intradermally into the shaven abdominal area four times at two-week intervals. For the challenge, 5 × 10^3^ 12B1 cells (approximately 10 TID_50_) in 0.2 mL PBS was administered s.c. into the flank area two weeks after the last vaccination. Mice were monitored three times a week for up to 90 days. When tumors reached the size of 400 mm^2^, the mice were humanely sacrificed.

### 2.3. In Vivo Depletion of CD4+ and CD8+ T Cell Populations

Mice were depleted of CD4+ or CD8+ T cell subsets, via intraperitoneal administration of anti-CD4+ and anti-CD8+ antibodies (both rabbit polyclonal, purified IgG fraction, Exbio, Prague, Czech Republic), respectively, 100 *μ*L/dose (as optimized in previous experiments). The antibodies were administered 3 days before the challenge, at the time of challenge (day 0) and then on days 7, 10, 17, and 24 after the challenge. The efficacy of depletion was monitored by flow cytometry two days after the second antibody dose and challenge. 

### 2.4. Flow Cytometric Analysis

After blocking nonspecific binding with rat anti-mouse CD16/32 (BD Pharmingen, San Diego, CA), splenocytes from antibody-treated and untreated mice were labelled with monoclonal antibodies FITC Rat Anti-Mouse CD4 (BD Pharmingen) or CD8a (mouse) PE (Exbio). The reduction of the respective cell subsets was determined via comparison of antibody-treated and control animals. To reveal other cell surface molecules, 12B1 cells were stained with monoclonal antibodies Anti-Mouse CD95 (APO-1/Fas) PE (eBioscience, San Diego, CA, USA), Anti-Mouse CD178 (Fas Ligand) PE (eBioscience), FITC Anti-Mouse I-A^d^ (subclass of MHC II) (Cedarlane Laboratories, Hornby, ON, Canada), and with corresponding isotype controls (eBioscience and Cedarlane). In addition, MHC class II expression by 12B1 cells was measured after 48 hrs incubation of cells in media supplemented with mouse recombinant IFN-*γ* (200 U/mL, Peprotech, London, UK). Cells were examined by a LSR Fortessa flow cytometer (BD Biosciences, San Jose, CA, USA). The results were analysed using FlowJo 7.6 software.

### 2.5. Statistical Analysis

For the analysis of survival, the Log-rank test was used. Calculations were done using Prism Software Version 5.0 (Graph-Pad Software, San Diego, CA). 

## 3. Results 

### 3.1. Monitoring In Vivo Depletion

**Figure 1:**
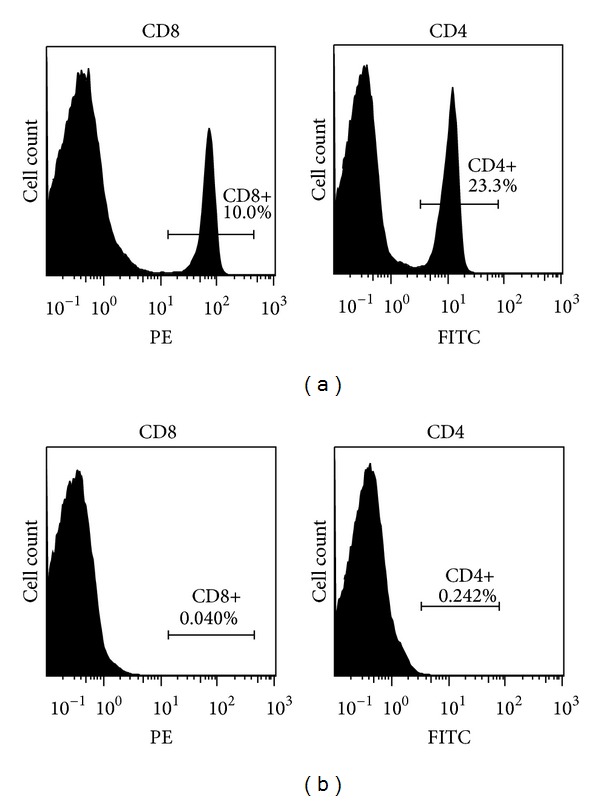
Flow cytometry analysis of splenocytes from animals (a) untreated and (b) treated with either rabbit polyclonal anti-CD4 or anti-CD8 antibody. Animals received two doses of depletion antibodies in three-day interval and were sacrificed two days after the second dose. The splenocytes were stained with rat monoclonal anti-CD4-FITC or anti-CD8-PE antibody.

The results of the depletion, obtained with splenocytes of animals sacrificed two days after the second dose of the respective antibody, are shown in Figure 1. Splenocytes from untreated animals served as controls. It can be seen that both CD8+ and CD4+ T cells were nearly completely depleted, indicating that the procedure used was highly effective.

### 3.2. Immunization Experiments

**Figure 2:**
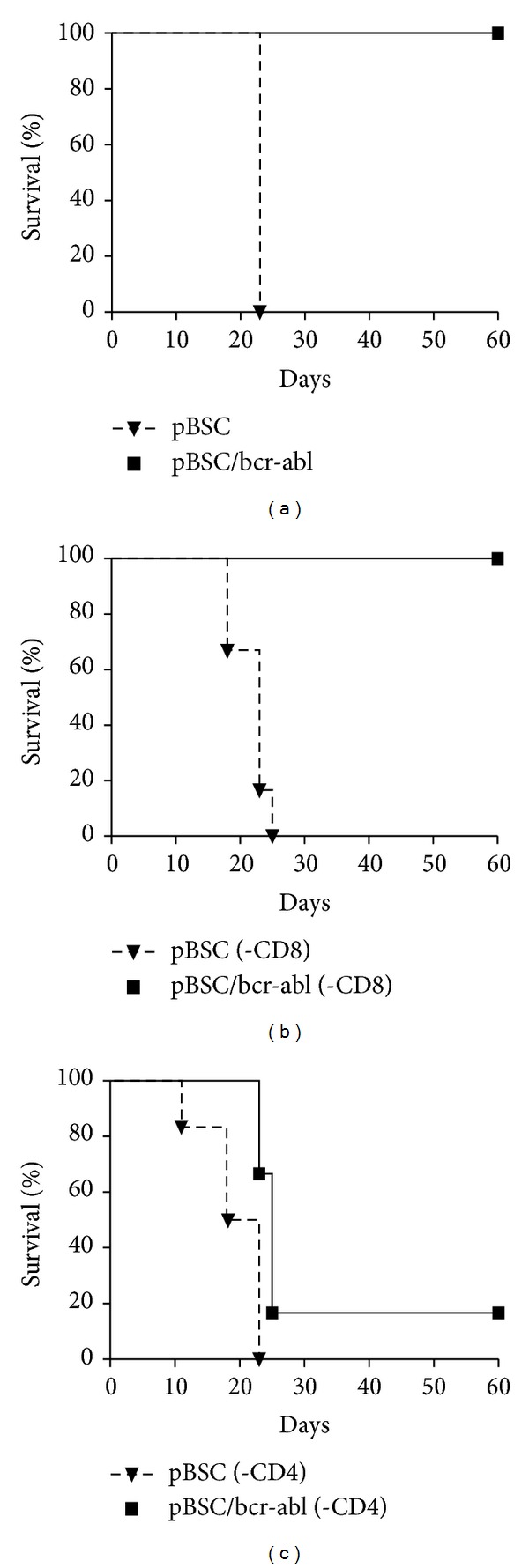
Survival of mice inoculated with empty pBSC or pBSC/bcr-abl plasmid and challenged with 12B1 cells. Six animals were in each group. (a) Nondepleted, (b) depleted of CD8+ T cells, and (c) depleted of CD4+ T cells.

The results of the immunization experiment are shown in Figure 2. The mice inoculated with the empty plasmid and subsequently treated or untreated with depletion antibodies developed tumors and died in all groups before day 25. Mice immunized with pBSC/bcr-abl plasmid and untreated with antibodies were fully protected against the challenge. Treatment with anti-CD8 antibody did not render the animals susceptible to tumor formation. On the other hand, 5 out of 6 immunized mice treated with anti-CD4+ antibody developed tumors and died before day 25. The difference between mice depleted of CD8+ T cells and those depleted of CD4+ T cells was statistically highly significant (*P* = 0.0054). The results indicated that CD4+ T cells and not CD8+ T cells acted as effectors in the present system. Similar data were obtained in the repeated experiment.

### 3.3. Expression of MHC Class II, Fas, and FasL Molecules on 12B1 Cells

**Figure 3:**
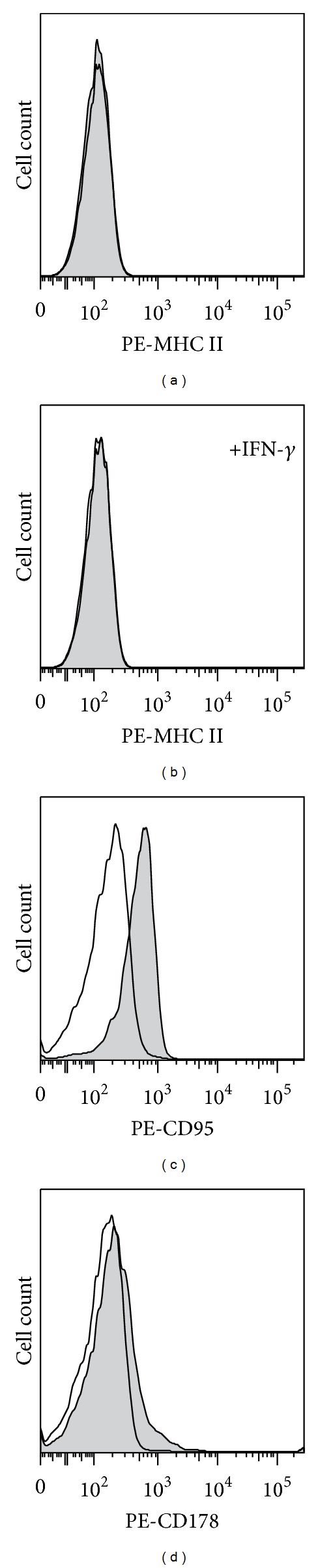
Flow cytometry analysis of 12B1 cells for the expression of MHC class II, Fas (CD95) and FasL (CD178), (a) cells treated with anti- MHC class II antibody, (b) cells cultivated in the presence of INF-*γ* and treated with MHC class II antibody, (c) cells treated with anti-Fas (CD 95) antibody, and (d) cells treated with anti-FasL (CD 178) antibody. Grey histograms indicate cells treated with the respective antibody. Empty histograms represent cells treated with the appropriate isotype controls.

In order to get information on the immune recognition molecules which might be involved in the anticancer effect, we used flow cytometry to measure levels of expression of MHC class II, Fas, and Fas L on the surface of 12B1 cells. The results are shown in Figure 3. It can be seen that these cells do not express the MHC class II molecules and that their expression was not induced by cultivation in the presence of INF-*γ*. On the other hand, they do express Fas and very low amounts of FasL.

## 4. Discussion

Quite recently it has been reported that SV40-induced tumors could be eliminated by specifically activated CD4+ T cells [15]. In those experiments, in which DNA vaccine was used for immunization, depletion of CD8+ T cells did not abolish the antitumor effect while depletion of CD4+ T cells rendered mice susceptible to tumor formation. Because of the timing of the immunization and subsequent administration of antibodies used for depletion, it was evident that CD4+ T cells played a critical role both in the activation and the effector phases. This was a rather surprising observation because in the previous experiments carried out by the same group of authors, in which they had immunized mice with recombinant SV40 T antigen, the antitumor effect was mediated through CD8+ T cells, with the possible contribution of antibody-dependent cell cytotoxicity (ADCC) [16, 17]. In addition, in those earlier experiments they had demonstrated that the CD4+ T cells were dispensable in the effector phase.

The present experiments revealed that the immunity against mouse bcr-abl-transformed 12B1 cells was mediated through the activity of CD4+ T cells. Their removal resulted in nearly complete abolishing of the anti-tumor immunity, while the depletion of CD8+ T cells did not impair it. Because of the timing of the depleting antibody administration, it seems clear that the CD4+ T cells were acting in the immune effector phase. The mechanisms involved are not clear at this writing. It is highly unlikely that the effect was directly mediated through MHC class II molecules. The presence of Fas on the 12B1 cells suggests but does not prove at this moment that Fas-FasL interaction was involved. However, it is also possible that the effects we observed were within the category of indirect effects induced by activated CD4+ T cells reported in other systems [1–3, 18, 19]. We also tried to establish the possible role of NK cells in the present undertaking. Using the same batch of antibody for depletion of NK cells, the results of repeated test were inconsistent, contributing—in a way—to the controversy about the role of these cells in tumor rejection as recently discussed [20, 21]. The equivocation of our results concerning NK cells does not seem to erode the conclusion that CD4+ T cells and not CD8+ T cells played the decisive role in tumor rejection in the present system.

The present results are strongly reminiscent of the previously mentioned data obtained in the SV40 system [15]. It may be of interest that in both their and our studies, DNA vaccines and BALB/c mice were employed. It will be the purpose of the future experiments to find out whether the vaccination constructs employed played any role in the differentiation of CD4+ T cells into effector cells, whether the mouse strain mattered, and whether the effects we observed were associated with the presence of human p210^bcr-abl^ in the mouse system or with the nature of the tumor cells expressing this protein, the key element in the pathogenesis of chronic myeloid leukaemia (CML). There are some other indications that CD4+ T cells may play the role of effectors in the immune reactions in CML. For example, it has been reported that CD4+ T cell clones isolated from several leukemic patients after bone marrow transplantation (BMT) were cytotoxic for leukemic myeloid cells but not for the other cells or cell lines [22]. It has also been demonstrated that HLA-DR restricted CD4+ T cell clones were cytotoxic for CML cells which processed and presented exogenous antigen (PPD); this led the respective authors to the conclusion that allogeneic donor CD4+ T cells might be sufficient for the induction of graft-versus-leukaemia (GvL) effect [23]. This seems to be in line with the results of another study in which infusion of donor CD4+ T lymphocytes depleted of CD8+ T lymphocytes resulted in GvL effect in a large proportion of patients [24]. In a more recent study, all four T cell clones isolated from CML patients after BMT, which had exhibited cytotoxicity for autologous tumor cells, were immunophenotyped as CD4+CD8− cells [25]. Also immune responses against autologous tumor cells in imatinib-treated CML patients were dominated by CD4+ T cells [26]. On the other hand, there are observations militating against the role of activated CD4+ T cells in eliminating leukemic cells. Thus, it has been shown that the CD4+ T cells responding to exposure to p210^bcr-abl^-derived peptides in a HLA-restricted manner did not exhibit cytotoxic activity [27] and that CD4+ T cell clones isolated from healthy HLA-DRB1 individuals and reactive with p210^bcr-abl^-derived peptides paradoxically enhanced the number of CML cell colonies when cocultivated with CML cells obtained from HLA-DRB1 positive patients [28]. In brief, there is now no really convincing evidence on an important or even decisive role for CD4+ T cells as effectors in anti-CML immunity. At this moment, speculations on the role of CD4+ cells have a character of a daring but testable hypothesis.

## 5. Conclusions

Without respect to the underlying mechanisms, the present observation provides some more ammunition for the present interest in the role for CD4+ T cells in effective phase of antitumor immune responses. The results of this study support the concept that CD8+ T cells do not command the antitumor immunity in all instances. Definitely, the role for CD4+ T cells as effectors in antitumor immunity should be further intensively investigated, because—due to the complex biology of these cells—many issues still remain unexplained. The identification and characterization of their subclass acting as cytotoxic effectors, if it really exists as suggested [5, 29], may provide a boost for the future research aimed at a better understanding of the determinants of tumor immunogenicity and novel immunotherapeutic options.
